# Unlocking metabolic flexibility in heart failure with preserved ejection fraction: Bridging fundamental mechanisms to clinical innovation

**DOI:** 10.1016/j.isci.2025.113471

**Published:** 2025-09-01

**Authors:** Junyan Xia, Yibing Nong, Jun Teng, Shafeeq A. Mohammed, Jing Liu, Yanting Pang, Sarah Costantino, Frank Ruschitzka, Nazha Hamdani, Mahmoud Abdellatif, Qian Lin, Francesco Paneni

**Affiliations:** 1 Department of Cardiology, Dongzhimen Hospital Affiliated to Beijing University of Chinese Medicine, Beijing 100700, China; 2 Center for Translational and Experimental Cardiology (CTEC), Department of Cardiology, University Hospital Zurich and University of Zürich, 8952 Zurich, Switzerland; 3 Center for Cardiometabolic Science, Division of Environmental Medicine, Department of Medicine, School of Medicine, University of Louisville, Louisville, KY 40202, USA; 4 Department of Respiration, Dongzhimen Hospital Affiliated to Beijing University of Chinese Medicine, Beijing 100700, China; 5 Department of Cardiology, Dongfang Hospital Affiliated to Beijing University of Chinese Medicine, Beijing 100078, China; 6 Department of Cardiology, University Heart Center, University Hospital Zurich and University of Zurich, 8091 Zurich, Switzerland; 7 Institute of Physiology, Department of Cellular and Translational Physiology, Ruhr University, Bochum 44791, Germany; 8 Institut für Forschung und Lehre (IFL), Molecular and Experimental Cardiology, St-Josef Hospital, Ruhr University, Bochum 44791, Germany; 9 Department of Cardiology, St-Josef Hospital, Ruhr University, 44791 Bochum, Germany; 10 HCEMM-SU Cardiovascular Comorbidities Research Group, Center for Pharmacology and Drug Research & Development, Department of Pharmacology and Pharmacotherapy, Intézet címe Semmelweis University, 1089 Budapest, Hungary; 11 Department of Physiology, University Maastricht, 6211 LK Maastricht, the Netherlands; 12 BioTechMed-Graz, Graz, Styria 8010, Austria; 13 Division of Cardiology, Medical University of Graz, Graz, Styria 8010, Austria

**Keywords:** Cardiovascular medicine, Human metabolism, Metabolic flux analysis

## Abstract

Heart failure is often described as a condition of “energy depletion.” However, in heart failure with preserved ejection fraction (HFpEF), particularly when associated with metabolic conditions such as obesity and diabetes, the heart may face a state of fuel overload. This fuel overload disrupts mitochondrial function, leading to the heart’s inability to effectively adjust substrate utilization in response to variations in nutritional status, energy substrate availability, and hemodynamic load, resulting in loss of metabolic flexibility and subsequent adverse effects on cardiac function and structure. Thus, an in-depth analysis of the role of metabolic flexibility in the pathophysiology of HFpEF could pave the way to addressing this clinical challenge. This review addresses: (1) the alterations in metabolic flexibility observed in cardiometabolic disease and HFpEF; (2) the implications of metabolic flexibility in the staging, classification, diagnosis, and prognosis of HFpEF; and (3) current HFpEF therapeutic strategies that improve myocardial metabolic flexibility.

## Introduction

With the aging population and the growing prevalence of obesity, hypertension, and diabetes, the incidence of heart failure with preserved ejection fraction (HFpEF) has increased significantly. A meta-analysis based on echocardiographic screening of the general population indicates that the prevalence of heart failure in individuals older than 65 years is 11.8%, with more than three-quarters of these cases classified as HFpEF.^1^ The hospitalization rate, length of hospital stay, and quality of life in patients with HFpEF are comparable to those in patients with heart failure with reduced ejection fraction (HFrEF).^2^ Moreover, studies have shown that the 5-year and 10-year mortality rates of HFpEF are 47% and 74%,^3^ respectively. HFpEF has thus emerged as a critical public health concern.

Unlike the traditional view that the failing heart is an “engine running out of fuel” in HFpEF, where patients often have comorbid metabolic conditions such as obesity and diabetes, the failing heart is thought to be exposed to a state of “fuel overload,” with mitochondria being inundated by excess substrates such as fatty acids (FAs), glucose, and amino acids.^4^ However, myocardial energy reserves in HFpEF patients are not increased but paradoxically reduced.^5^^,^^6^ This fuel excess leads to inefficient mitochondrial metabolism and impaired substrate switching, causing incomplete substrate utilization for energy production and subsequent storage in ectopic depots.^7^ This phenomenon is described as a loss of metabolic flexibility in myocardial energy substrate utilization. The progression of HFpEF represents the shift from adaptive to maladaptive metabolic remodeling, where the failing heart loses its ability to switch to the most efficient energy-producing fuels, with the accumulation of toxic metabolic intermediates driving the disease process.

**Figure 1:**
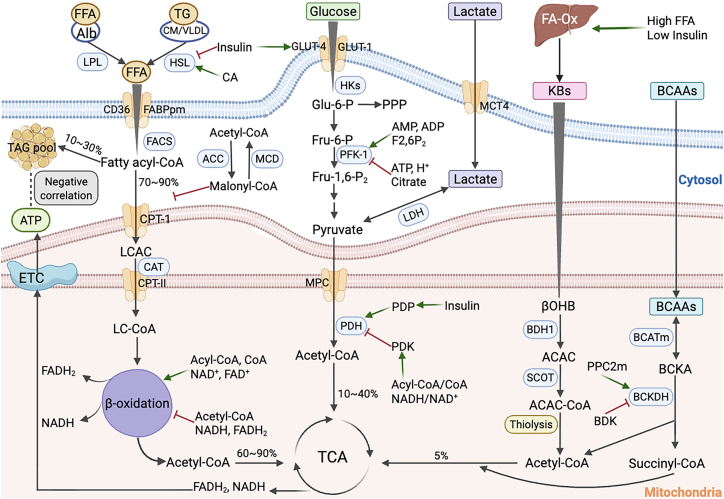
Overview of myocardial metabolic flexibility A healthy heart exhibits high flexibility in fuel utilization and is capable of switching substrates for acetyl-CoA and ATP production depending on the availability of energy substrates, hormonal signals, oxygen concentration, and physiological needs. The heart can utilize a variety of substrates—such as fatty acids, glucose, lactate, ketones, and amino acids—for energy conversion. Metabolic flexibility relies not only on the sensing, uptake, transport, storage, and utilization of nutritional substrates but also on the synthesis, degradation, or activity regulation of key proteins involved in metabolic pathways. Alb, albumin; LPL, lipoprotein lipase; HSL, hormone-sensitive lipase; TG, triglyceride; CM, chylomicron; VLDL, very low-density lipoprotein; CA, catecholamines; ACC, acetyl-CoA carboxylase; MCD, malonyl-CoA decarboxylase; PPP, pentose phosphate pathway; BDK, branched-chain ketoacid dehydrogenase kinase; PPC2m, protein phosphatase C2m; FFA, free fatty acids; FA-Ox, fatty acids oxidation; FACS, fatty acyl-CoA synthetase; TAG, triglyceride; LCAC, long-chain acylcarnitine; CAT, carnitine acyltransferase; LC-CoA, long-chain acyl-CoA; ETC, electron transport chain; ATP, adenosine triphosphate; HKs, hexokinases; Glu-6-P, glucose 6-phosphate; Fru-6-P, fructose 6-phosphate; Fru-1,6-P2, fructose 1,6-bisphosphate; PFK-1, phosphofructokinase-1; LDH, lactate dehydrogenase; PDH, pyruvate dehydrogenase; PDK, PDH kinase; PDP, PDH phosphatase; TCA, tricarboxylic acid; KBs, ketone bodies; βOHB, β-hydroxybutyrate; BDH1, β-Hydroxybutyrate dehydrogenase 1; ACAC, acetoacetate; AcAc-CoA, acetoacetyl-CoA; BCAAs, branched-chain amino acids; BCATm, mitochondrial BCAAs aminotransferase; BCKA, branched-chain α-keto acid; BCKDH, branched-chain α-keto acid dehydrogenase. Created in BioRender. XIA, J. (2025) https://BioRender.com/m11k924.

In 1999, Kelley et al. first introduced the concept of “metabolic flexibility,” referring to an organism’s ability to select the most efficient energy substrate in a given physiological environment.^8^ This regulation of substrate switching can be described as the organism’s acute or chronic adaptation to short-term or long-term changes in energy demands.^4^ A healthy heart exhibits high flexibility in fuel utilization (Figure 1), dynamically switching substrates for acetyl-CoA and ATP production in response to energy substrate availability, hormonal signals, oxygen concentration, and physiological demands. Thus, the heart is capable of switching between a variety of substrates, such as FAs, glucose, lactate, ketones, and amino acids, for energy production, rather than simultaneously increasing (or decreasing) the utilization rates of multiple substrates.^9^ Under different physiological conditions, the levels of energy substrate metabolism change to meet energy demands or fluctuations in oxygen availability. Metabolic flexibility is not a simple “on-off” phenomenon; instead, it involves finely tuned adjustments regulated by precise mechanisms.^4^ Metabolic flexibility relies on the sensing, uptake, transport, storage, and utilization of nutritional substrates, as well as the synthesis, degradation, or activity regulation of key proteins within metabolic pathways. Therefore, metabolic flexibility can be understood as an organism’s adaptive response to metabolic demands.

Kelley proposed measuring the respiratory quotient (RQ) through the euglycemic-hyperinsulinemic clamp to assess metabolic flexibility.^8^^,^^10^ This approach involves using a whole-room indirect calorimeter (respiration chamber) to measure changes in RQ from a fasting state to fuel-stimulated conditions (e.g., clamp, meal tests, exercise, catecholamine infusion) as δRQ = RQ(t)−fasting RQ.^11^^,^^12^ δRQ reflects the efficiency of ATP production under specific fuel conditions but does not reveal substrate preferences or changes in metabolic pathways in the organism or myocardium. Another common method to determine myocardial metabolic flexibility involves the use of isotopic tracers to measure metabolic flux and substrate preference.^13^ Positron emission tomography (PET) is frequently used for this purpose, with the main advantages being the flexibility in the design of radiotracers and its quantitative capabilities,^14^^,^^15^ allowing imaging of metabolic processes within the field of view. However, PET requires specially designed tracers targeting specific pathways (e.g., uptake or oxidation), and its production and imaging costs are high. Additionally, it is not well suited for measuring the abundance of multiple fuel metabolic pathways.^16^^,^^17^ Metabolomics, which examines small molecules and metabolites (e.g., amino acids, FAs, and carbohydrates), is a more comprehensive approach, capable of comparing changes in metabolic substrates and assessing metabolic flexibility.^18^^,^^19^^,^^20^^,^^21^ Given the challenges of directly detecting changes in myocardial intracellular metabolic pathways, some researchers have adopted an arteriovenous sampling method combined with metabolomics to infer myocardial substrate uptake and utilization indirectly.^22^^,^^23^ The limitations of metabolomics include its limited spatial resolution, as metabolites may diffuse across tissues and throughout the body, making it challenging to distinguish interactions between different cell types within the microenvironment. Thus, metabolomics must be integrated with proteomics and transcriptomics to identify changes in relevant pathways and protein targets, thereby reflecting adaptive changes in metabolic pathways.^19^^,^^24^^,^^25^ Metabolic remodeling precedes and can trigger and sustain functional and structural remodeling of the heart, and this process is reversible.^26^ Continuous imaging studies of cardiac metabolic activity *in vivo* have demonstrated this.^27^ Early identification of the progression from adaptive to maladaptive metabolic flexibility, and timely intervention to halt this process, may help attenuate or potentially reverse the deterioration of cardiac function. Therefore, investigating the role of metabolic flexibility in the development of HFpEF may provide insights into correcting metabolic abnormalities. This review summarizes the alterations in myocardial metabolic flexibility in cardiometabolic disease and HFpEF; highlights the clinical implications of metabolic flexibility in the risk stratification, phenotyping, diagnosis, and prognosis of HFpEF; and reviews current HFpEF therapeutic strategies that improve myocardial metabolic flexibility.

## Myocardial metabolic flexibility in cardiometabolic disease

Overweight, obesity, and associated insulin resistance are fundamental contributors to the development and progression of cardiometabolic diseases.^28^ A clinical study based on four community cohorts found that obesity and related cardiometabolic characteristics, including insulin resistance, significantly increase the risk of future HFpEF, particularly in women.^1^ Thus, the metabolic phenotype is one of the critical characteristics of HFpEF.

Obesity results from a positive energy balance, characterized by an increase in white adipose tissue, which primarily stores excess metabolic energy. In contrast, brown adipose tissue is rich in mitochondria and actively oxidizes substrates to generate heat. Brown adipose tissue activity helps prevent hyperglycemia and hyperlipidemia by oxidizing circulating metabolic substrates, whereas white adipose tissue expansion and their mitochondrial fragmentation and dysfunction due to obesity is associated with systemic metabolic changes, including hyperglycemia, insulin resistance, and dyslipidemia.^29^ Under stress, inflammatory pathways in white adipocytes activate several Ser/Thr kinases, directly impairing insulin receptor signaling in adipocytes and leading to local insulin resistance.^30^ This insulin resistance reduces glucose utilization and increases lipolysis, leading to hyperglycemia and hypertriglyceridemia in obese individuals. Local infiltration of immune cells, increased pro-inflammatory cytokines, and ectopic lipid deposition in peripheral tissues further reduce insulin sensitivity.^31^ Persistent hyperglycemia and reduced insulin sensitivity eventually lead to β-cell exhaustion in the pancreas, driving the development of type 2 diabetes mellitus (T2DM).

Obesity is predominantly associated with elevated plasma free fatty acid (FFA) levels. Hyperlipidemia and hyperinsulinemia stimulate the transport of FFAs into cardiomyocytes. Elevated FFA levels inhibit glycogen synthase activity and increase pyruvate dehydrogenase kinase expression through PPARα-mediated transcriptional activation, leading to pyruvate dehydrogenase inactivation and reduced glucose utilization and oxidation.^32^ When FFA delivery exceeds the oxidative capacity of cardiomyocytes, lipid accumulation can lead to lipotoxicity,^33^ triggering a form of cardiomyopathy, characterized by cardiac hypertrophy and diastolic dysfunction, ultimately progressing to cardiometabolic HFpEF.^34^ When triglyceride storage in subcutaneous adipose tissue reaches maximum capacity with limited ability for further expansion, lipids accumulate in ectopic depots such as skeletal muscle and the liver. Ectopic fat depots release more inflammatory mediators and exhibit greater macrophage infiltration than subcutaneous adipose tissue,^35^ closely associating them with metabolic abnormalities, insulin resistance, T2DM, and cardiovascular disease.^33^ Thus, metabolic inflexibility and fat deposition may create a self-reinforcing vicious cycle.

Insulin shifts fuel utilization in skeletal muscle from FA oxidation during fasting to glucose utilization in the fed state.^36^ Patients with insulin resistance typically rely less on FA oxidation during fasting, and FA oxidation does not seem to decrease during insulin infusion.^4^ The metabolic characteristics of insulin resistance include reduced FA oxidation, decreased capacity for glucose uptake and oxidation, and mitochondrial dysfunction, characterized by limited or abnormal mitochondrial respiratory protein expression and activity.^37^ Patients with cardiometabolic diseases overly rely on carbohydrate-derived energy and have limited ability to switch between carbohydrate and fat oxidation.^38^ Metabolic inflexibility is ultimately attributed to poor mitochondrial respiratory capacity and related dysfunction.^11^^,^^39^ Mitochondria adjust their morphology based on nutrient availability, thereby regulating oxidative phosphorylation activity and substrate preference.^40^ In the skeletal muscle of insulin-resistant patients, mitochondrial expression of PGC1α and its downstream targets is reduced,^41^ and the expression of the mitochondrial outer membrane fusion protein mitofusin-2 is decreased, affecting mitochondrial dynamics and quality control.^42^ Studies have shown that long-term high-fat feeding in rodents or prolonged exposure of myotubes to FAs can reduce the expression of nuclear-encoded mitochondrial genes.^43^

Hence, patients with cardiometabolic diseases experience low-grade chronic inflammation and ectopic fat accumulation due to caloric excess, which affects insulin sensitivity and leads to metabolic inflexibility characterized by poor switching capacity between carbohydrate and FA oxidation, potentially related to mitochondrial dysfunction.

## Myocardial metabolic flexibility in the HFpEF myocardium

Myocardial metabolic inflexibility is a prominent feature in HFpEF patients,^44^^,^^45^ where the efficiency and flexibility of energy substrate utilization affect ATP production, subsequently causing myocardial functional and structural abnormalities. Understanding energy substrate utilization shifts during HFpEF development can provide insights into its pathophysiological mechanisms, potentially identifying therapeutic strategies to combat HFpEF and addressing the current paucity of effective treatment options.

### Fatty acid metabolism

Metabolic substrates differ in their efficiencies and oxygen requirements. Effective regulation of energy substrate utilization is crucial for restoring homeostasis under various conditions, including fed or fasted states, hypoxia, and the presence of obesity, diabetes, or aging. Under both physiological and pathological conditions, FA metabolism remains the primary energy source for the heart.^46^^,^^47^ When FA metabolism decreases and alternative fuels fail to compensate for the ATP shortfall, normal cardiomyocyte function is compromised. Obesity and insulin resistance are generally believed to skew energy metabolism toward FA oxidation. Increased circulating FFAs enhance transcriptional programs for fat uptake and breakdown, whereas insulin-dependent myocardial glucose uptake is impaired.^34^ Because HFpEF patients often have comorbidities such as T2DM, obesity, and insulin resistance, it is widely understood that their myocardial FA β-oxidation rate is increased.^46^^,^^47^^,^^48^ In a “two-hit” mouse model of HFpEF induced by high-fat diet (HFD) and N[w]-Nitro-L-arginine methyl ester (L-NAME), perfusion of radiolabeled energy substrates into isolated hearts also showed increased FA oxidation rates.^49^ However, this increase in FA oxidation did not enhance overall cardiac ATP production; instead, it was disrupted. Possible reasons include mitochondrial overload and incomplete β-oxidation, both of which impair mitochondrial function and metabolic flexibility.^50^ Impaired FA oxidation significantly contributes to insulin resistance.^50^ Another perspective suggests that FA oxidation varies with the stage of HFpEF development: it may be unaffected or enhanced in the early stages,^51^ whereas FA uptake and oxidation are significantly impaired in the later stages.^52^ While metabolomic profiles cannot reveal dynamic changes in metabolism, they can provide a snapshot of alterations in metabolic pathways at specific points during HFpEF progression. A clinical study analyzing metabolomic changes in plasma and myocardial biopsy tissue from HFpEF patients found that, despite significant obesity and diabetes, myocardial FA metabolites (medium- and long-chain acylcarnitines [MLACs]) were lower in HFpEF compared to HFrEF, with only two medium-chain acylcarnitines elevated in plasma. This finding was consistent with the low expression of FA-metabolism-related genes revealed by RNA sequencing.^45^ Other studies have shown that, although the proportion of obese and diabetic patients was lower in the HFpEF group, plasma MLAC levels were higher compared to control groups.^53^ MLACs, which are structurally esterified to carnitine, facilitate the transfer of medium- and long-chain FAs to mitochondria for β-oxidation. MLACs are typically transient but accumulate during inefficient FA oxidation.^50^ In a single-arm study of 13 patients with hypertensive HFpEF, plasma metabolomics analysis showed increased acylcarnitine metabolites,^54^ consistent with other findings^53^^,^^55^ and suggesting impaired FA oxidation. In the “two-hit” HFpEF mouse model, both palmitoylcarnitine- and pyruvate-induced respiration were reduced in isolated heart mitochondria, indicating upstream defects in pyruvate and FA metabolism. The mechanism may involve hyperacetylation of key enzymes in this pathway, with sirtuin 3 downregulation and NAD deficiency leading to excessive acetylation of mitochondrial proteins.^56^ In another non-obese feline HFpEF model induced by mild aortic constriction, the abundance of acylcarnitines in myocardial metabolites increased, and transcriptomic data also indicated impaired FA oxidation.^57^ Additionally, the accumulation of long-chain acylcarnitines is toxic to sarcoplasmic phospholipids and may interact with various ion channels, leading to arrhythmias.^58^ Abnormal lipid metabolism is central to HFpEF pathogenesis. Measures that promote FA oxidation can reduce cardiac lipid accumulation and alleviate myocardial fibrosis, ultimately improving HFpEF.^59^^,^^60^

### Carbohydrate metabolism

The ATP deficit resulting from impaired FA metabolism may prompt an upregulation of alternative metabolic pathways, though such compensatory mechanisms could be maladaptive. A clinical study involving cardiac biopsies from 38 HFpEF patients revealed that myocardial levels of ketone bodies (KBs) and TCA cycle metabolite were lower. In contrast, pyruvate levels were elevated compared to those in HFrEF and control groups.^45^ RNA sequencing demonstrated reduced expression of essential genes involved in glucose metabolism in HFpEF patients, including the glucose transporter 1 (SLC2A1), which is crucial for glucose uptake. These findings collectively suggest impaired alternative fuel utilization and compromised glucose oxidation pathways in HFpEF patients.^45^^,^^61^ Multiple studies support this conclusion.^44^^,^^51^ One clinical study further demonstrated elevated expression of GLUT1 and glucose levels in HFpEF myocardium, accompanied by significantly reduced levels of glycolytic metabolites and their associated enzymes (such as glucose-6-phosphate, fructose-1,6-bisphosphate, hexokinase, and phosphofructokinase), possibly linked to glycogen accumulation, further underscoring metabolic inflexibility in HFpEF myocardium.^44^ In the Dahl salt-sensitive (DSS) rat model of HFpEF, subjected to a high-salt diet, serial assessments of energy metabolism at 3, 6, and 9 weeks post-induction demonstrated a progressive increase in cardiac glycolysis, which paralleled the worsening of diastolic dysfunction (as measured by the echocardiographic E′/A′ ratio). The uncoupling of glycolysis and glucose oxidation led to significant increases in proton and lactate levels.^52^ Increased proton concentrations and a drop in pH reduced troponin I sensitivity to calcium and inhibited slow calcium currents, further weakening myocardial contractility.^62^ Additionally, ATP was expended in removing these protons and maintaining sodium and calcium homeostasis, reducing cardiac efficiency and contributing to functional decline.^52^^,^^63^ The decoupling of glycolysis and glucose oxidation also independently activated myocardial remodeling pathways, resulting in cardiac hypertrophy and promoting heart failure progression.^52^ In a feline HFpEF model, induced by mild aortic constriction, multi-omics analysis revealed a metabolic shift from oxidative phosphorylation to glycolysis.^57^ Metabolomic changes observed at 4 months, compared to 1-month post-aortic constriction, provided further evidence for a highly enriched Warburg effect, indicating a preference for glycolysis over oxidative phosphorylation under aerobic conditions.^57^ However, other preclinical studies challenged this notion that glycolysis is enhanced in HFpEF.^49^^,^^51^

### Ketone body metabolism

KBs have been proposed as an efficient auxiliary fuel for the failing heart, given their ability to rapidly enter cells and cross mitochondrial membranes along a concentration gradient without requiring rate-limiting transport proteins.^9^ Compared to glucose and FAs, KBs generate more ATP per mole of oxygen consumed, indicating their potentially superior energetic efficiency. However, findings regarding circulating KBs levels in HFpEF patients remain inconsistent. One clinical study reported significantly elevated serum levels of AcAc and β-OHB in patients with HFpEF compared to those with HFrEF,^53^ suggesting enhanced systemic ketone availability. In contrast, other studies have shown no substantial increase in either cardiac or circulating ketone levels in the HFpEF patients,^45^ indicating that endogenous ketone utilization may be limited. Notably, a recent randomized, double-blind, crossover clinical trial demonstrated that exogenous ketone supplementation improved cardiac output, reduced ventricular stiffness and filling pressures, and significantly lowered pulmonary capillary wedge pressure (PCWP) during peak exercise in patients with HFpEF and T2DM.^64^ These functional benefits were recapitulated in a HFpEF mouse model induced by aging, long-term high-fat feeding, and desoxycorticosterone pivalate challenge, where ketone therapy alleviated diastolic dysfunction, potentially via modulation of the mitochondria-inflammation circuit.^48^ These findings suggest that although endogenous ketone metabolism may not be upregulated in HFpEF, targeted metabolic modulation via exogenous ketone therapy could represent a promising therapeutic avenue, warranting further exploration.

### Amino acid metabolism

The oxidation of branched-chain amino acids (BCAAs), including leucine, isoleucine, and valine, primarily represents amino acid oxidation in the heart. Although the contribution of BCAA oxidation to ATP production in the heart is relatively small (approximately 2%),^47^ BCAAs and their metabolic intermediates play significant roles in cellular signaling pathways. A clinical study demonstrated that myocardial BCAA levels were highest in HFpEF patients compared to HFrEF patients and controls, although their BCAA metabolite levels were lower.^45^ RNA sequencing results indicated decreased expression of key enzymes in the BCAA metabolic pathway—such as BCKDHB (branched-chain α-ketoacid dehydrogenase E1 subunit β), DBT (dihydrolipoamide branched-chain transacylase E2), and SLC25A44 (responsible for transporting BCAAs from the cell membrane to mitochondria)—suggesting impaired BCAAs oxidation in the HFpEF heart.^45^^,^^61^ These genes are also regulated by PPARα, a major regulator of FA metabolism.^65^ BCAAs are oxidized into acetyl-CoA and propionyl-CoA, both of which fuel the TCA cycle. When oxidation is impaired, BCAAs accumulate in the circulation, influencing signaling pathways such as the leucine-mediated activation of mTOR, a key regulator of cell growth, cardiac hypertrophy, and insulin resistance.^66^ Studies have shown that BCAAs promote insulin resistance, with mTOR as one of the key mechanisms.^67^ Therefore, impaired BCAA metabolism and accumulation may limit the heart’s fuel supply and exacerbate insulin resistance, promoting the development of HFpEF. Alterations in amino acid metabolism also play a significant role in HFpEF. Previous studies have shown that myocardial and circulating amino acid levels are elevated in HFpEF patients compared to controls.^45^^,^^53^^,^^68^ Additionally, baseline levels of asparagine/aspartate are negatively correlated with clinical HFpEF scores,^68^ with some amino-acid-derived metabolites also associated with echocardiographic markers of diastolic dysfunction.^55^ In animal models, significant changes in amino acids, including increased BCAAs levels, were observed early in HFpEF development.^57^ Further research found that amino acids closely related to extracellular matrix remodeling and collagen formation (e.g., glutamine, glycine, serine) were negatively correlated with myocardial tissue fibrosis, suggesting that alterations in amino acid metabolism may contribute to the development of interstitial fibrosis during HFpEF progression.^57^

**Figure 2:**
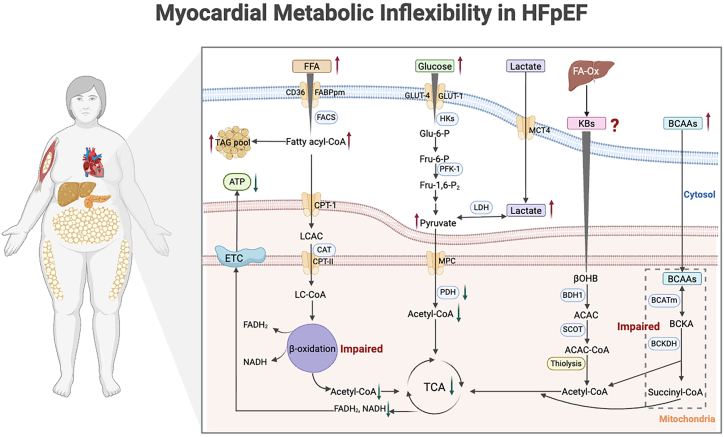
Myocardial metabolic inflexibility in HFpEF The heart in HFpEF exhibits significant metabolic inflexibility, characterized by impaired glucose oxidation and the uncoupling of glycolysis from glucose oxidation, leading to the accumulation of lactate and protons. The mismatch between fatty acid uptake and oxidation exacerbates lipotoxicity in cardiomyocytes. Additionally, insufficient ketone body oxidation and disrupted BCAA oxidation pathways further limit energy production, worsen insulin resistance, and contribute to the progression of HFpEF. HFpEF, heart failure with preserved ejection fraction; FFA, free fatty acid; FA-Ox, fatty acid oxidation; FACS, fatty acyl-CoA synthetase; TAG, triglyceride; LCAC, long-chain acylcarnitine; CAT, carnitine acyltransferase; LC-CoA, long-chain acyl-CoA; ETC, electron transport chain; ATP, adenosine triphosphate; HKs, hexokinases; Glu-6-P, glucose 6-phosphate; Fru-6-P, fructose 6-phosphate; Fru-1,6-P2, fructose 1,6-bisphosphate; PFK-1, phosphofructokinase-1; LDH, lactate dehydrogenase; PDH, pyruvate dehydrogenase; TCA, tricarboxylic acid; KBs, ketone bodies; βOHB, β-hydroxybutyrate; BDH1, β-hydroxybutyrate dehydrogenase 1; ACAC, acetoacetate; AcAc-CoA, acetoacetyl-CoA; BCAAs, branched-chain amino acids; BCATm, mitochondrial BCAAs aminotransferase; BCKA, branched-chain α-keto acid; BCKDH, branched-chain α-keto acid dehydrogenase. Created in BioRender. XIA, J. (2025) https://BioRender.com/p58o271.

Thus, the heart in HFpEF exhibits significant metabolic inflexibility (Figure 2), characterized by impaired glucose oxidation and the uncoupling of glycolysis from glucose oxidation, leading to the accumulation of lactate and protons. The mismatch between FA uptake and oxidation exacerbates lipotoxicity in cardiomyocytes. Additionally, insufficient ketone body oxidation and disrupted BCAA oxidation pathways further limit energy production and worsen insulin resistance. This metabolic dysregulation not only contributes to energy deficiency but also promotes the progression of HFpEF through the disruption of epigenetic modifications.

## Application of myocardial metabolic flexibility in staging and phenotyping of HFpEF

The staging of HFpEF remains inadequately defined. According to the 2023 JACC statement, HFpEF can be categorized into three stages: stage A, which includes the presence of risk factors for HFpEF (analogous to stage A in the American College of Cardiology/American Heart Association [ACC/AHA] chronic heart failure staging); stage B, which refers to the cardiovascular remodeling phase of preclinical HFpEF (similar to stage B in the ACC/AHA chronic heart failure staging); and stage C, which represents the often underdiagnosed phase of HFpEF (corresponding to the clinical HFpEF stages C and D).^69^ Diagnosing HFpEF primarily relies on a history of symptoms such as dyspnea and fatigue, evidence of congestion, and a preserved ejection fraction (EF) of ≥50%.^70^ However, detecting objective evidence of congestion, particularly in outpatient settings, can be challenging.^71^ Studies indicate that approximately one-third of HFpEF patients have plasma BNP levels below the clinical threshold typically used for diagnosing heart failure,^72^ a situation prevalent in obese HFpEF patients.^73^ Right heart catheterization remains the gold standard for diagnosing HFpEF from a hemodynamic perspective, particularly by identifying elevated PCWP at rest or during exercise. Nevertheless, the invasive nature of this procedure limits its widespread application, complicating the diagnosis of HFpEF. Interestingly, emerging research on energy metabolism has identified specific metabolic panels that correlate with different stages and subtypes of HFpEF, offering a promising avenue for improved diagnosis, staging, and phenotyping of the condition.^74^

### Metabolic flexibility in HFpEF staging

**Figure 3:**
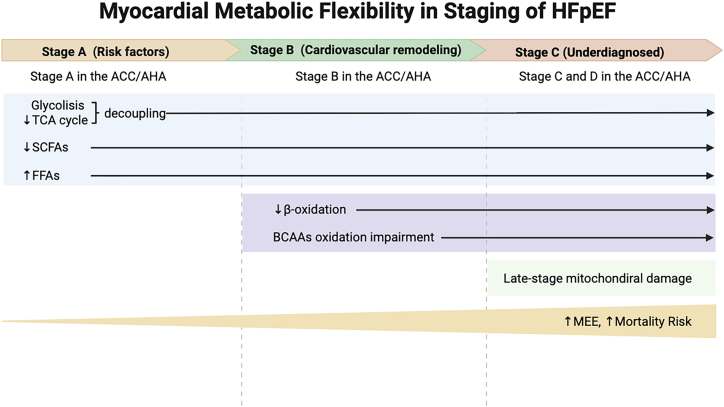
Myocardial metabolic flexibility in the staging of HFpEF Stage A of HFpEF is characterized by glycolysis-TCA cycle uncoupling, an increase in free fatty acids, and a deficiency in short-chain fatty acids. As HFpEF progresses, mitochondrial dysfunction leads to incomplete fatty acid β-oxidation and impaired BCAA oxidation, which can be monitored during stage B of HFpEF. Stage C focuses on markers associated with late-stage mitochondrial failure. Despite the progressive loss of myocardial fuel flexibility in HFpEF, myocardial energy expenditure continues to increase as the disease advances, along with a corresponding rise in mortality risk. HFpEF, heart failure with preserved ejection fraction; SCFAs, short-chain fatty acids; FFAs, free fatty acids; TCA, tricarboxylic acid; BCAAs, branched-chain amino acids; MEE, myocardial energy expenditure. Created in BioRender. XIA, J. (2025) https://BioRender.com/r83n531.

The adaptability and maladaptation of cardiac metabolic flexibility are reflected in the dynamic changes observed in cardiac structure and function. Preclinical studies of HFpEF have demonstrated a rapid increase in glycolysis preceding the onset of diastolic dysfunction.^52^ This increase is associated with the decoupling of glycolysis from the TCA cycle and the subsequent accumulation of protons, even without impaired FA oxidation.^51^^,^^52^ The glycolysis-TCA cycle decoupling, which contributes to the rapid progression of HFpEF and its dependence on glycolysis, is further underscored by the accumulation of protons and glutamate, alongside a reduction in alanine and lactate—metabolites considered early indicators of HFpEF.^74^ Short-chain FAs, produced through the anaerobic fermentation of dietary fiber by the gut microbiota, have been linked to hypertension, hypertrophy, fibrosis, and diabetes^75^ and are also associated with HFpEF.^76^ Some researchers suggest early assessment of these metabolites in HFpEF may be warranted.^74^ Monitoring the levels of metabolites associated with early stages (A and B) of HFpEF—such as protons, alanine, glutamate, and short-chain FAs—along with the duration and intensity of comorbidities, could provide valuable insights. In stage C, monitoring β-oxidation, BCAA impairment, or early markers of HFrEF (e.g., LCAC, BCAAs, bile salts, and trimethylamine) could be beneficial. In contrast, stage D should focus on markers of late-stage mitochondrial failure (e.g., KBs, succinate, phospholipids, lipids, serine, arginine, ADMA, SDMA, MMA, TMAO). This stratified approach could enhance our understanding of HFpEF progression.^74^ A cross-sectional study found that higher concentrations of lipid- and amino-acid-derived metabolites, particularly those associated with mitochondrial damage (e.g., butyrylcarnitine and N-formylmethionine), could serve as early biomarkers of subclinical left ventricular diastolic dysfunction.^55^ The flexibility in energy substrate switching determines the myocardium’s total energy usage. Despite the loss of fuel flexibility in HFpEF, myocardial energy expenditure (MEE) is not decreased as expected. In a study by Yu Wang et al., 125 HFpEF patients were divided into two groups based on the degree of diastolic dysfunction: group A (8 ≤ E/e′ ≤ 15) and group B (E/e′ > 15). They calculated MEE using echocardiographic parameters and related formulas, finding that while myocardial energy substrate metabolism remains near normal in the early stages of HFpEF, MEE increases in the later stages, correlating with a higher mortality risk.^77^ This suggests that patients with a more severe HFpEF experience increased myocardial oxygen and energy consumption, possibly alongside heightened neurohormonal activity, further depleting cardiac energy reserves. The abnormal MEE observed also reflects the progressive deterioration of contractile function in HFpEF patients,^52^ indicating that MEE could serve as a clinical monitoring parameter and a biomarker for prognostic risk stratification, particularly in patients with diastolic dysfunction within the gray zone (8 ≤ E/e′ ≤ 15). Eduardo Anguita et al. investigated energy metabolism markers in the plasma of HFpEF patients during stable and decompensated phases, finding that decompensated patients were characterized by lower levels of lactate dehydrogenase B and histidine, elevated levels of lactate and formate, and reduced mitochondrial DNA copy number. Conversely, the stable phase was characterized by a marked decrease in formate levels and high mitochondrial DNA copy numbers.^78^ Lactate dehydrogenase B, which catalyzes the conversion of lactate to pyruvate, when downregulated, can lead to sustained reduced oxidative phosphorylation and enhanced glycolysis. Formate may also increase glycolysis rates,^79^ reflecting a more robust glycolytic phenotype during the acute phase of HFpEF and significant energy metabolism inflexibility. Clearly defining metabolic flexibility and its driving mechanisms during HFpEF progression is crucial (Figure 3), as it will guide efforts to mitigate metabolic abnormalities and clarify their role in disease progression.

### Metabolic flexibility in HFpEF phenotyping

**Figure 4:**
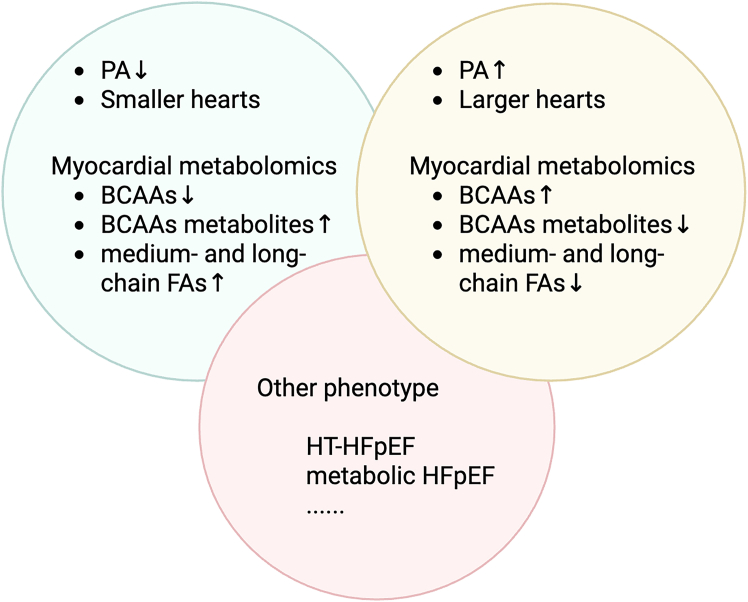
Myocardial metabolic flexibility in phenotyping of HFpEF Using non-negative matrix factorization, myocardial metabolomic profiles of all HFpEF patients were grouped, revealing two distinct clusters. One cluster is characterized by pulmonary hypertension and enlarged hearts, associated with elevated BCAA levels, lower BCAA catabolites, and medium- to long-chain fatty acids. The other cluster presents with near-normal pulmonary artery pressure and heart size, lower BCAA levels, higher BCAA catabolites, and medium- to long-chain fatty acids. Additionally, based on comorbidities, metabolic-specific panels for phenotypes such as HT-HFpEF and metabolic HFpEF remain to be fully elucidated. HFpEF, heart failure with preserved ejection fraction; PA, pulmonary artery; FA, fatty acid; BCAAs, branched-chain amino acids; HT, hypertension. Created in BioRender. XIA, J. (2025) https://BioRender.com/f92l574.

HFpEF is a multisystem, clinically heterogeneous disease strongly associated with risk factors such as aging, female sex, obesity, diabetes, hypertension, and physical inactivity.^1^^,^^69^ It has been proposed that the failure of multiple drug trials to improve outcomes in HFpEF patients may be due to underlying phenotypic heterogeneity.^80^ Recently, research has focused on using machine learning to integrate clinical variables, physical characteristics, laboratory data, electrocardiogram (ECG) parameters, and echocardiographic findings to biologically phenotype HFpEF patients into categories such as obesity/metabolic, elderly hypertension and vascular aging, and right heart dysfunction/pulmonary vascular disease.^61^^,^^81^ However, these phenotypic classifications are difficult to define by a single concept or metric, posing a significant challenge in studying HFpEF subtypes. Metabolic characterization studies offer a promising approach to elucidate the existence of distinct subgroups among HFpEF patients (Figure 4). High-throughput technologies have identified unique gene expression profiles related to metabolic pathways in these patients.^61^ For instance, a study by Hahn et al. performed myocardial metabolomics after categorizing HFpEF patients based on clinical characteristics but found no significant differences. However, when all HFpEF patients were grouped using non-negative matrix factorization, three distinct subgroups emerged, two of which showed the greatest degree of separation, with significant contribution of BCAAs metabolism as a biomarker.^45^ One subgroup displayed clinical features of pulmonary hypertension and enlarged hearts, accompanied by high levels of methylhistidine, proline, and isoleucine (BCAAs) and persistently low levels of BCAA metabolites and medium- and long-chain FAs. In contrast, the other group exhibited near-normal pulmonary artery pressure and heart size, along with low levels of methylhistidine, proline, and isoleucine (BCAAs) and persistently high levels of BCAAs metabolites and medium- and long-chain FAs. These findings suggest that the pulmonary hypertension subgroup is characterized by inhibited FA oxidation and defects in amino acid and BCAA oxidation pathways. In contrast, the other subgroup’s metabolism tends toward normal.^45^ Furthermore, researchers have identified heterogeneity within the HFpEF population by differentiating metabolic response patterns induced by exercise training (ET). Key distinguishing metabolites include spermidine, lyso PC a C18:0, lyso PC a C18:1, 29 glycerophospholipids, and 7 sphingolipids. The clinical characteristics of these subgroups were associated with baseline perceived well-being (SF-36 score) and changes in low-density lipoprotein levels but were not related to cardiopulmonary function, ventilatory function, or echocardiographic parameters.^82^ The biological roles of these differential metabolites are primarily associated with FA oxidation, energy storage, autophagy, and cellular signaling. It remains to be seen whether this classification approach can improve predictions of responses to ET and other treatments. Metabolomics analysis of specific HFpEF patient groups, such as those with hypertensive HFpEF, has revealed increased plasma acylcarnitine metabolites. A sodium-restricted diet has been shown to improve cardiac function by increasing the availability of short-chain acyl residues.^54^ In DSS rats, a model of hypertensive HFpEF, plasma and left ventricular free carnitine levels decreased when fed a high-salt diet.^83^ These findings suggest that hypertensive HFpEF patients may experience not only limited FA transport but also impaired FA oxidation pathways, leading to energy shortages. Thus, further research is warranted to exploit metabolic characteristics to subgroup HFpEF patients.

## Diagnostic and prognostic value of myocardial energy metabolites in HFpEF

As previously mentioned, the clinical diagnosis of HFpEF remains challenging, often leading to delays in treatment and management, which may contribute to the high mortality rate in this patient population. However, studies on energy metabolism offer promising avenues for improving both the diagnosis and prognostic assessment of HFpEF. For instance, a clinical study employed metabolomic fingerprinting to identify novel biomarkers specific to HFpEF. This study identified a panel of four metabolites (acetate, 2-hydroxybutyrate, momordin acylcarnitine, and phosphatidylcholine [C40:4]) that, when combined with BNP, were able to distinctly differentiate HFpEF from HFrEF patients, achieving an area under the receiver operating characteristic curve (ROC AUC) of 0.981. Another metabolite panel consisting of octanoylcarnitine, arginine, and sphingomyelin (C20:2), when combined with NT-proBNP, could distinguish HFpEF patients from non-HF controls, with an ROC AUC of 0.942. Notably, the accuracy of these newly identified metabolite panels, as indicated by ROC AUC values, was significantly higher than that of existing protein biomarkers.^53^ Furthermore, targeted metabolomics analysis of plasma samples from participants in the PRESERVED-HF trial revealed that increased levels of BCAA/BCKA and ketone-related metabolites, along with decreased levels of medium- and long-chain acylcarnitines, were associated with improved outcomes in HFpEF patients.^84^ These findings underscore the potential application of energy metabolites in enhancing the diagnosis and prognosis of HFpEF.

Momordin acylcarnitine and octanoylcarnitine are crucial coenzymes in FA metabolism, facilitating the transport of FAs into mitochondria. Similar to findings in other studies, carnitine deficiency has been suggested as a prognostic predictor for HFpEF.^85^ Butyrylcarnitine, another acylcarnitine family member, has been positively correlated with left ventricular relaxation.^55^ Additionally, N-formylmethionine, an initiator of protein synthesis in eukaryotic cell mitochondria, has shown a positive correlation with left ventricular filling pressure, indicating its potential diagnostic value in HFpEF alongside butyrylcarnitine.^55^ Trimethyllysine, a lysine residue derivative and precursor of carnitine, plays a role in the FA β-oxidation process. A clinical study in an Asian population demonstrated that higher plasma trimethyllysine levels are positively associated with HFpEF and an increased risk of cardiovascular events, underscoring its significant diagnostic and prognostic value for HFpEF.^86^ Research by Wang et al. identified short-chain dicarboxylic lactam metabolites (products of short-chain FA metabolism) and the asparagine/aspartate ratio as biomarkers linked to poor clinical outcomes in HFpEF.^68^ These findings highlight the critical role of FA oxidation substrates and their metabolites in diagnosing and prognostically assessing HFpEF, likely due to their importance in myocardial energy supply. However, the clinical application of these metabolite panels for diagnosing HFpEF remains to be validated, necessitating further research to establish their utility.

## Strategies for improving myocardial metabolic flexibility in HFpEF

Impaired myocardial metabolic flexibility is a key contributor to the reduced energy efficiency in HFpEF. As a result, enhancing metabolic flexibility has emerged as a potential therapeutic target for HFpEF. Here, we summarize current strategies that effectively improve metabolic flexibility in HFpEF myocardium and explore how these interventions modulate substrate utilization, promote energy production, and enhance cardiac function to slow the progression of HFpEF (Table 1).

**Table 1:** Strategies for improving myocardial metabolic flexibility in HFpEF

Strategy	Role in treating HFpEF	Mechanism on metabolic flexibility
Exercise training	improves aerobic capacity and quality of life in HFpEF patients^87^^,^^88^^,^^89^	corrects defects in fatty acid oxidation and skeletal muscle mitochondrial function, promoting the recovery of metabolic flexibility^82^^,^^90^
SGLT2 inhibitors	reduces the combined risk of cardiovascular death or heart failure hospitalization in HFpEF patients, regardless of diabetes presence^91^^,^^92^; improves exercise capacity and quality of life^93^	lowers blood glucose and insulin resistance^94^; promotes a metabolic shift favoring fatty acid oxidation, with physiologic increases in ketone availability that may support myocardial energetic adaptation^95^^,^^96^^,^^97^
GLP-1 receptor agonists	improves physical function and heart failure symptoms in HFpEF patients^98^	reduces blood glucose and body weight^99^; further research is needed to clarify the exact impact on metabolic flexibility
Trimetazidine	shows no significant effect on pulmonary capillary wedge pressure during exercise in HFpEF patients^100^	reduces mitochondrial fatty acid β-oxidation while enhancing glucose oxidation^101^
AdipoRon	in a “two-hit" HFpEF mouse model, reduces cardiac lipid accumulation, alleviates myocardial fibrosis, and improves cardiac function^59^	enhances fatty acid metabolism and improves insulin sensitivity^102^
NAD^+^ supplementation	diets rich in NAD+ precursors have been shown to reduce blood pressure, cardiovascular mortality, and all-cause mortality^6^	restores fatty acid and branched-chain amino acid metabolism, shifting energy metabolism from glycolysis to more energy-efficient oxidative processes^103^

HFpEF, heart failure with preserved ejection fraction.

### Exercise training

Physical inactivity and obesity are strongly associated with worse health outcomes and prognosis in HFpEF patients, and these factors may also significantly contribute to metabolic inflexibility.^12^ Regular physical exercise can enhance metabolic flexibility. ET has been shown to improve aerobic capacity and quality of life in HFpEF patients.^87^^,^^88^^,^^89^ Studies indicate moderate-intensity training is as effective as higher intensity training,^87^ but only high-intensity interval training has demonstrated the ability to induce molecular changes. These changes include increased mitochondrial enzyme activity, enhanced expression of mitochondrial oxidative phosphorylation complexes, significant reductions in muscle atrophy markers such as MuRF1 and Trim72, and improved satellite cell function, which is crucial for muscle repair and regeneration. However, these molecular benefits are sustained only with high adherence to the exercise regimen, as they tend to diminish when the regimen is transitioned to a home-based program.^104^ The EX-DHF-P trial (ISRCTN86879094) further confirmed the benefits of ET in HFpEF patients. Metabolic response analysis from this trial revealed that ET prevents the increase in acetylornithine and carnitine levels and prevents the decrease in three glycerophospholipids, suggesting that ET may improve defects in FA oxidation by modulating carnitine availability, thereby promoting the recovery of metabolic flexibility.^82^ ET has been shown to positively affect skeletal muscle mitochondrial function,^90^ which may contribute to the alleviation of exercise intolerance in HFpEF patients. A recent study that analyzed respiratory measurements of biopsy-derived muscle fiber bundles from HFpEF patients identified significant abnormalities in skeletal muscle mitochondrial function, including severely reduced maximal capacity and complex-I- and complex-II-associated respiration, which correlated with exercise intolerance.^105^ Two experimental studies using different animal models of HFpEF (ZSF1 and DSS rats) demonstrated that ET can prevent mitochondrial and functional damage^106^ and reduce reliance on glycolytic metabolism.^107^ In a “two-hit” HFpEF mouse model, ET was found to reverse the HFpEF myocardial phenotype by altering N6-methyladenosine (m6A) modifications.^108^ M6A is known to modify RNA methylation, and Gene Ontology and Kyoto Encyclopedia of Genes and Genomes analyses revealed that differentially methylated genes are related to myocardial energy metabolism, fibrosis, hypertrophy, and apoptosis.^108^ AMPK is thought to be a key regulator of the changes in metabolic flexibility induced by ET.^109^ However, the extent to which ET improves myocardial metabolic flexibility in HFpEF and the specific molecular mechanisms involved still require further research.

### SGLT2 inhibitors and glucagon-like peptide-1 receptor agonists

Initially developed as antihyperglycemic agents, SGLT2 inhibitors (SGLT2is) have shown significant cardiovascular benefits, independent of diabetic status, particularly in heart failure patients. These benefits include a marked reduction in the risks of heart failure hospitalization and cardiovascular mortality.^110^ The EMPEROR-Preserved and DELIVER trial results further demonstrated that SGLT2is can reduce the combined risk of cardiovascular death or heart failure hospitalization in patients with HFpEF, regardless of diabetes.^91^^,^^92^ Additionally, the PRESERVED-HF trial showed that SGLT2is improved patients’ health status, exercise capacity, and quality of life.^93^ These findings have driven new clinical guidelines, affirming the first-line role of SGLT2is in treating HFpEF.^69^^,^^110^

SGLT2i functions by inhibiting the expression of SGLT2 in the proximal renal tubules, which reduces renal glucose reabsorption and promotes urinary glucose excretion, thereby lowering blood glucose levels. Notably, their mechanism of action is independent of insulin and pancreatic β-cell function, allowing SGLT2is to reduce both blood glucose and glycated hemoglobin levels while enhancing β-cell functionality and decreasing insulin resistance.^94^ SGLT2i-induced reductions in plasma glucose and insulin levels are accompanied by increases in circulating FFAs and modest elevations in plasma KBs. This metabolic shift may promote enhanced lipid oxidation and provide ketones as an additional myocardial energy substrate. Recent studies suggest that the degree of ketosis induced by SGLT2is is generally within physiological ranges and may not be sufficient to independently drive major alterations in cardiac metabolism.^95^ However, preclinical evidence indicates that enhanced ketone oxidation may partially contribute to the cardioprotective effects of SGLT2is, as demonstrated in murine models of heart failure with impaired ketone utilization.^96^ The overall impact on myocardial substrate flexibility likely reflects a combined modulation of glucose, FA, and ketone metabolism.^97^ Beyond glycemic control, SGLT2is confer additional benefits, including lowering blood pressure, reducing body weight, decreasing triglyceride levels, and minimizing proteinuria.^111^ Experimental studies have shown that canagliflozin ameliorates myocardial hypertrophy, fibrosis, and left ventricular diastolic dysfunction in DSS rat models of HFpEF. The underlying mechanism is believed to involve activation of the AMPK/SIRT1/PGC-1α pathway, which reduces myocardial glucose metabolism, enhances FA metabolism and ketogenesis, restores myocardial metabolic flexibility, and decreases oxidative stress in the myocardium.^112^ In male Zucker diabetic fatty rat HFpEF models, dapagliflozin was found to reduce nitro-oxidative stress, pro-inflammatory cytokine levels, myocardial hypertrophy, and fibrosis, effectively preventing the onset of HFpEF through AMPK activation and inhibition of the mTOR pathway.^113^ Key metabolic pathways implicated include cholesterol and high-density lipoprotein particle metabolism, niacin and nicotinamide metabolism, arginine biosynthesis, and cAMP and PPAR signaling.^113^ However, a randomized placebo-controlled trial indicated that 12 weeks of dapagliflozin treatment did not significantly alter systemic metabolism, as reflected by circulating metabolites in HFpEF patients.^84^ This indicates that the metabolic mechanisms underlying its effects require further investigation. In addition, sotagliflozin, a dual SGLT1/2 inhibitor, has also shown promise in HFpEF. Preclinical studies have demonstrated that it ameliorates left atrial remodeling and calcium-mediated cellular arrhythmogenesis in a metabolic-syndrome-related rat model of HFpEF.^114^ These effects are associated with improved mitochondrial calcium buffering capacity, prevention of mitochondrial swelling, and reduced oxidative stress under glycolytic inhibition, suggesting that SGLT1 inhibition may help restore cardiomyocyte metabolic and ionic homeostasis. While direct evidence for significant alterations in myocardial substrate oxidation with SGLT1 inhibition remains limited, these findings point to a potentially complementary metabolic mechanism that warrants further exploration in HFpEF.

Glucagon-like peptide-1 (GLP-1) receptor agonists, such as semaglutide, are gaining increasing attention due to their potential benefits in reducing body weight and major adverse cardiovascular events (MACEs) in diabetic patients.^99^ The pooled analysis of the STEP-HFpEF and STEP-HFpEF DM trials demonstrated that, compared to placebo, semaglutide significantly improved physical function and heart failure symptoms in HFpEF patients.^98^ The SELECT trial further confirmed the efficacy of semaglutide in reducing MACE in patients with atherosclerotic cardiovascular disease and overweight or obesity.^115^ Preclinical studies on HFpEF have also shown that semaglutide positively affects cardiac metabolism.^116^ In a mouse model of angiotensin II (AngII)-induced diastolic dysfunction, liraglutide improved ventricular function, normalized myocardial levels of citric acid cycle and pentose phosphate intermediates, and increased cardiac protein turnover through enhanced amino acid uptake and translation.^117^ Additionally, liraglutide increased myocardial acetoacetate levels, altered short- and long-chain acylcarnitine profiles, and reduced FA oxidation capacity, thus indicating a shift in substrate utilization. In line with these findings, another study using hyperpolarized ^13^C and ^31^P magnetic resonance spectroscopy showed that liraglutide treatment normalized TCA cycle metabolism and improved phosphocreatine-to-ATP ratio while restoring myocardial energetics in obese rats with diastolic dysfunction.^118^ Liraglutide was also found to enhance myocardial glucose oxidation in diabetic mice through indirect mechanisms involving increased insulin levels,^119^ while a clinical trial with the GLP-1RA albiglutide reported modest improvements in peak oxygen consumption, despite minimal changes in myocardial glucose or oxygen uptake.^120^ These findings highlight that GLP-1RAs may influence myocardial energy metabolism through multiple interrelated pathways. However, the precise role of GLP-1RAs in modulating myocardial metabolic flexibility in HFpEF remains to be fully elucidated and warrants further investigation in both clinical and mechanistic studies.

### Trimetazidine and AdipoRon

Trimetazidine is a well-established drug recognized globally for its ability to improve energy metabolism and is widely approved for the symptomatic treatment of chronic stable angina.^121^ It works by inhibiting mitochondrial long-chain 3-ketoacyl-CoA thiolase, the final enzyme in the FA β-oxidation pathway, reducing mitochondrial FA β-oxidation while simultaneously enhancing glucose oxidation.^101^ Additionally, trimetazidine has been shown to lower myocardial glycolysis rates, thereby reducing the accumulation of hydrogen ions and lactate in the cytoplasm, which helps prevent adverse cardiac events such as calcium overload.^122^ Furthermore, trimetazidine can improve insulin resistance, increase myocardial glucose uptake, and enhance glucose oxidation.^123^ In HFrEF patients, three months of trimetazidine treatment improved the myocardial PCr/ATP ratio by 33%.^124^ Clinical benefits observed with trimetazidine include improved symptoms, enhanced quality of life, increased exercise tolerance, reduced NT-proBNP levels, and improved both systolic and diastolic cardiac function. Moreover, it has been associated with reduced hospitalization rates and lower all-cause mortality.^121^ Experimental studies have further demonstrated that trimetazidine can improve both systolic and diastolic function in obese heart failure mouse models.^125^ However, recent evidence from a randomized, double-blind, placebo-controlled crossover trial (DoPING-HFpEF) indicated that trimetazidine had no significant effect on PCWP during various levels of exercise and did not improve myocardial energy homeostasis in HFpEF patients.^100^ This suggests that trimetazidine may not confer the expected clinical benefits in HFpEF. On the other hand, AdipoRon, an oral synthetic adiponectin receptor agonist, enhances FA metabolism and insulin sensitivity.^102^ In a “two-hit” HFpEF mouse model, AdipoRon was shown to reduce cardiac lipid accumulation, alleviate myocardial fibrosis, and improve cardiac function.^59^ These findings emphasize a critical aspect of the HFpEF pathogenesis—dysregulated FA metabolism—and highlight the necessity of exploring myocardial metabolic intervention strategies.

### NAD^+^ supplementation therapy

NAD^+^ supplementation therapy is a promising treatment approach for HFpEF, though it has not yet been specifically validated in clinical trials for this condition. However, substantial evidence from several preclinical studies supports its potential efficacy.^6^^,^^56^ NAD exists in both oxidized (NAD^+^) and reduced (NADH) forms and functions as a key electron carrier coenzyme in energy metabolism processes, including glycolysis, the TCA cycle, and oxidative phosphorylation. NAD^+^ also serves as a substrate for PARPs, sirtuins, and CD38, thereby contributing to DNA repair, epigenetic regulation, post-translational modifications, and metabolic adaptation to changes in nutritional status.^126^ Given the heart’s high energy demands, the cardiovascular system is particularly vulnerable to disturbances in NAD^+^ metabolism. NAD^+^ depletion can impair mitochondrial FA β-oxidation and oxidative phosphorylation, reducing myocardial bioenergetic efficiency and compromising cardiac pump function.

Maintaining NAD^+^ homeostasis requires dietary intake of NAD^+^ precursors (such as nicotinamide, niacin, and nicotinamide riboside, collectively known as vitamin B3). For a comprehensive explanation of NAD^+^ biosynthesis and metabolism pathways, refer to the relevant reviews.^46^ NAD^+^ supplementation therapy has demonstrated cardiovascular protective effects in clinical trials, with no significant adverse effects reported.^127^ Research indicate that HFpEF is associated with reduced cardiac NAD^+^ levels. Diets rich in naturally occurring NAD^+^ precursors have been shown to reduce blood pressure, cardiovascular mortality (including death due to heart failure, myocardial infarction, and sudden cardiac death), and all-cause mortality risk.^6^ Preclinical studies have found lower NAD^+^ levels in the hearts and livers of ZSF1 obese rat HFpEF models and in diet-induced metabolic syndrome.^6^ Oral supplementation of NAD^+^ precursors, such as nicotinamide, has improved diastolic dysfunction in rodent HFpEF models associated with aging (in 2-year-old C57BL/6J mice), hypertension (in DSS rats), or cardiometabolic syndrome (in ZSF1 obese rats).^6^ Furthermore, indole-3-propionic acid, a metabolite produced by gut microbiota from dietary tryptophan, was reduced in the plasma of HFpEF patients and model mice.^103^ Studies suggest that indole-3-propionic acid supplementation can restore levels of nicotinamide, NAD^+^/NADH, and SIRT3 in the heart by inhibiting NNMT, thereby alleviating diastolic dysfunction, metabolic remodeling, oxidative stress, inflammation, gut microbiota dysbiosis, and gut epithelial barrier damage in HFpEF model mice.^103^ Metabolomic analysis revealed that nicotinamide primarily increased levels of unsaturated FAs, acylcarnitines, and 3-hydroxybutyric acid, while reducing BCAAs and their keto-acid derivatives and restoring glycolytic intermediates. TCA cycle metabolites and oxidative-phosphorylation-related genes were also upregulated. This suggests that NAD^+^ supplementation in HFpEF model mice restored FA and BCAA metabolism, shifting energy metabolism from glycolysis to more energy-efficient oxidative processes, thereby enhancing metabolic flexibility and reversing the metabolic inflexibility characteristic of HFpEF. Additionally, nicotinamide restored myocardial energy reserve capacity (as indicated by increased left ventricular ATP/ADP and PCr/ATP ratios) and improved obesity-related parameters.^6^ In the “two-hit” HFpEF mouse model, defects in pyruvate and FA metabolism were identified in cardiomyocytes. Oral nicotinamide riboside supplementation corrected cardiac NAD^+^ deficiency, restored palmitoylcarnitine-mediated mitochondrial respiration, and improved cardiac remodeling, diastolic function, exercise capacity, and pulmonary congestion.^56^ Whether NAD^+^ supplementation can emerge as a promising therapeutic approach for HFpEF requires validation through high-quality clinical studies.

## Conclusion

Cardiac metabolic flexibility is essential for the capacity of the heart to rapidly generate ATP and maintain normal systolic and diastolic functions in response to fluctuations in nutritional status, substrate availability, and hemodynamic load. In HFpEF, the frequent co-existence of metabolic disorders leads to mitochondrial overload and the toxic accumulation of substrates and intermediates, depriving the heart of flexibility in energy substrate utilization. Metabolic changes not only affect ATP production but can also lead to global disruptions in the epigenome, influencing epigenetic modifications. For example, metabolites such as acetyl-CoA and S-adenosylmethionine serve as donors for histone acetylation and methylation, respectively, while succinate and fumarate can inhibit DNA and histone demethylases. These epigenetic alterations, in turn, can affect the metabolic pathways of energy substrates. Our recent study demonstrated that the histone methyltransferase SETD2 is upregulated in the left ventricular myocardium of HFpEF patients with metabolic dysregulation. SETD2 promotes triglyceride accumulation and lipotoxic damage by regulating the transcription of the lipotoxicity-related gene SREBP1. In contrast, selective deletion of SETD2 reduces lipid accumulation and improves metabolic remodeling and functional impairment in HFpEF hearts.^128^ These disorders ultimately results in impaired cardiac function and structural changes. Future research should focus on further characterizing the alterations in metabolic flexibility observed in HFpEF, investigating the impact of these changes on cardiac function, and elucidating the underlying mechanisms. Additionally, it is important to analyze how metabolic flexibility is regulated across different HFpEF phenotypes or stages. Such insights will be critical in guiding the development of targeted interventions to improve metabolic health in HFpEF.

## Acknowledgments

This work is supported by the 10.13039/501100001711Swiss National Science Foundation (grant no: 310030_197557 and grant no: 320030-236150), the 10.13039/501100004362Swiss Heart Foundation, the 10.13039/501100012654Olga Mayenfisch Foundation, the 10.13039/501100013161Swiss Life Foundation, the Kurt und Senta-Hermann Stiftung, the 10.13039/501100008464EMDO Stiftung, and the Schweizerische Diabetes-Stiftung (to F.P.). J.X. is supported by the China Scholarship Council scholarship (grant no: 202306550021). Q.L. is supported by the High-Level Traditional Chinese Medicine Discipline Construction Project of National Administration of Traditional Chinese Medicine (zyyzdxk-2023253) and the Qihuang Scholar Program (2021).

## Author contributions

J.X. and J.T. researched data for the article. F.P., Q.L., N.Y., and J.X. discussed the content of the manuscript and provided critical intellectual feedback. J.X. wrote the manuscript. All the authors reviewed/edited the manuscript before submission.

## Declaration of interests

The authors declare no competing interests. During the preparation of this work, we used Chat GPT-4o to improve readability and language. After using this tool, we reviewed and edited the content as needed and take full responsibility for the content of the publication.
